# Metaplastic breast carcinoma versus triple-negative breast cancer: distinct metastatic patterns and inferior overall survival in a matched retrospective cohort study

**DOI:** 10.3389/fonc.2026.1932260

**Published:** 2026-09-22

**Authors:** Rui Yang, Yuelan Ma, Rui Chang, Shuang Ma, Hong Li, Dahai Chai, Jinping Li

**Affiliations:** 1 The First Clinical Medical College, Ningxia Medical University, Yinchuan, China; 2 Department of Surgical Oncology, General Hospital of Ningxia Medical University, Yinchuan, China

**Keywords:** clinicopathological characteristics, metaplastic breast carcinoma, recurrence and metastasis, survival outcomes, triple-negative breast cancer

## Abstract

**Background:**

Metaplastic breast carcinoma (MBC) is an uncommon invasive breast cancer subtype with marked morphological diversity. Many MBCs are managed within the triple-negative breast cancer (TNBC) framework, yet their clinical course may not mirror that of non-metaplastic TNBC. We therefore compared clinicopathological features, recurrence and metastatic patterns, and survival outcomes in patients with MBC and those with non-metaplastic TNBC.

**Methods:**

We reviewed 27 patients with pathologically confirmed MBC who were treated at the Cancer Hospital of Ningxia Medical University General Hospital from January 2015 to December 2023. From patients with non-metaplastic TNBC treated in the same period, 81 controls were randomly selected at a 1:3 ratio and matched by age and year of diagnosis. We assessed clinicopathological variables, immunohistochemical findings, treatment patterns, recurrence and metastasis, disease-free survival (DFS), and overall survival (OS). Survival was evaluated with Kaplan-Meier curves and log-rank tests.

**Results:**

The two cohorts were comparable in age and menopausal status. Relative to TNBC, MBC more often presented as grade III disease (88.9% vs. 28.7%, *P* < 0.001) and T3–T4 disease (33.3% vs. 11.1%, *P* = 0.007), while nodal involvement was less common (25.9% vs. 51.9%, *P* = 0.019). Proliferative activity was greater in MBC, with a higher Ki-67 index (54.0 ± 16.3% vs. 43.5 ± 22.0%, *P* = 0.011) and a higher proportion of tumors with Ki-67 ≥ 30% (100.0% vs. 81.5%, *P* = 0.020). During follow-up, recurrence or metastasis (25.9% vs. 8.6%, P = 0.041) and distant metastasis (22.2% vs. 2.5%, P = 0.003) occurred more often in MBC. DFS was numerically shorter in MBC but was not statistically different (*P* = 0.200), whereas OS was significantly poorer (*P* = 0.038).

**Conclusion:**

In this matched cohort, MBC showed a profile of high-grade histology, greater tumor burden, active proliferation, limited nodal spread, and frequent distant metastasis. The results indicate that MBC should not be treated simply as another presentation of TNBC, but as a high-risk entity requiring careful assessment of metastatic risk.

## 1 Introduction

Breast cancer is a highly heterogeneous disease with diverse molecular and histological subtypes (1, 2). Advances in subtype-based diagnosis and treatment have improved outcomes for many patients. However, rare histological variants remain difficult to manage because of their low incidence, limited evidence base, and distinct biological behavior (3, 4). MBC is an uncommon malignant breast tumor in which epithelial tumor cells acquire squamous, spindle-cell, chondroid, osseous, or other mesenchymal-like differentiation (5, 6). This morphological diversity can complicate both pathological recognition and clinical decision-making (7). Although MBC represents only a small fraction of breast cancers, published series have repeatedly described large primary tumors, high histological grade, high proliferative activity, and substantial risks of relapse and metastasis (8).

Because most MBCs lack estrogen receptor (ER), progesterone receptor (PR), and human epidermal growth factor receptor 2 (HER2) expression, they are often classified clinically within the TNBC spectrum (9). However, this receptor-based classification does not fully reflect the distinct pathology of MBC. Unlike conventional TNBC, MBC is defined by lineage plasticity and divergent differentiation, features that are not reflected by receptor status alone (10). It may also express basal-like or myoepithelial markers such as cytokeratin 5/6 (CK5/6), epidermal growth factor receptor (EGFR), tumor protein p63 (p63) (11, 12). Previous studies further suggest that MBC often presents with larger primary tumors, less frequent lymph node involvement, and a greater tendency toward distant hematogenous spread, indicating biological behavior that differs from conventional TNBC.

Treatment evidence specific to MBC remains limited, largely because the disease is rare and difficult to evaluate in prospective randomized trials (5). In routine practice, therapeutic decisions are usually extrapolated from treatment strategies for invasive breast cancer or TNBC, including surgery, chemotherapy, radiotherapy, and, in selected cases, immunotherapy or targeted therapy (13, 14). Although pragmatic, this approach may not fully account for the unique histological and biological characteristics of MBC. Several retrospective studies have reported that MBC responds less favorably to conventional chemotherapy than non-metaplastic TNBC, particularly in the neoadjuvant setting, where pathologic complete response rates appear to be low (7, 15). Survival outcomes have also been reported to be poorer in patients with MBC, although results vary across cohorts because of differences in sample size, stage distribution, treatment patterns, and follow-up duration (16–18). These uncertainties highlight the need to compare MBC directly with non-metaplastic TNBC in well-defined clinical cohorts. Such comparisons may help clarify whether MBC carries distinct risks of recurrence, distant metastasis, and death, and may provide a stronger basis for risk stratification and individualized treatment planning.

In this retrospective study, we conducted a matched retrospective cohort study to compare MBC with non-metaplastic TNBC. By evaluating clinicopathological characteristics, immunohistochemical profiles, treatment patterns, recurrence and metastatic patterns, and survival outcomes, we aimed to clarify whether MBC represents a clinically distinct and higher-risk entity within the TNBC spectrum.

## 2 Methods

### 2.1 Study population

This single-center retrospective cohort included patients with MBC who were diagnosed and treated at the Cancer Hospital of Ningxia Medical University General Hospital between January 2015 and December 2023. Diagnosis was confirmed pathologically using surgical specimens or needle biopsy. Patients with non-metaplastic TNBC treated during the same period formed the source population for controls. Controls were randomly selected at a 1:3 ratio and matched by diagnosis year and age, yielding 27 patients with MBC and 81 patients with TNBC. Exclusion criteria were male breast cancer, bilateral breast cancer, concurrent malignancy, completion of primary treatment elsewhere, incomplete pathological records, or unavailable follow-up data. Clinicopathological comparisons and OS analysis used the full cohort. For DFS analysis, patients with distant metastasis at initial diagnosis and those who did not undergo surgery were excluded.

### 2.2 Pathological data and immunohistochemical assessment

The extracted variables were age, menopausal status, histological type, histological grade, T stage, N stage, M stage, overall stage, histological subtype, and immunohistochemical results. Tumor staging was determined according to the 8th edition of the Union for International Cancer Control (UICC) TNM classification. MBC subtypes followed the WHO classification of breast tumors and institutional pathology reports. ER and PR positivity required nuclear staining in ≥ 1% of tumor cells. HER2 positivity was assigned for IHC 3+ or fluorescence *in situ* hybridization (FISH) amplification; IHC 0/1+ or IHC 2+ without FISH amplification was classified as HER2-negative. Ki-67 was dichotomized at 30%, with Ki-67 ≥ 30% considered high expression. CK5/6 and EGFR status were abstracted as positive or negative from the original reports.

### 2.3 Treatment modalities and follow-up

Treatment variables comprised surgery, neoadjuvant chemotherapy, postoperative adjuvant chemotherapy, radiotherapy, and endocrine therapy. Surgery was classified as modified radical mastectomy, mastectomy with sentinel lymph node biopsy, breast-conserving surgery, or no surgery. Neoadjuvant chemotherapy referred to systemic treatment before surgery, including chemotherapy with or without immunotherapy. Postoperative adjuvant chemotherapy mainly represented adjuvant chemotherapy. Radiotherapy included chest wall or breast irradiation and regional nodal irradiation, and was recorded as yes or no. Because endocrine therapy was relevant only for hormone receptor-positive tumors, it was not statistically compared in the TNBC group.

Follow-up information was collected from outpatient records, inpatient records, and telephone interviews. The data cutoff date was June 30, 2026. DFS was calculated from surgery to local recurrence, regional recurrence, distant metastasis, death, or last follow-up. OS was calculated from diagnosis to death from any cause or last contact. Breast cancer-specific death was defined as death attributable to breast cancer progression, recurrence, or distant metastasis. Treatment-related deaths and deaths unrelated to breast cancer were not classified as breast cancer-specific deaths. Event-free patients were censored at their most recent follow-up.

### 2.4 Statistical analysis

Analyses were conducted in R software version 4.3.0. Continuous variables are presented as mean ± standard deviation or median (interquartile range), depending on distribution, and were compared with the t-test or Mann–Whitney U test. Categorical variables are presented as counts and percentages and were compared with the χ² test or Fisher’s exact test when appropriate. The proportions of breast cancer-specific deaths and overall deaths between the MBC and TNBC cohorts were compared using Fisher’s exact test. Median follow-up duration was estimated using the reverse Kaplan–Meier method, and follow-up distributions between groups were compared using the log-rank test. Kaplan–Meier estimates were used for survival curves, with log-rank testing for group comparisons. Two-sided *P* values < 0.05 were considered statistically significant.

### 2.5 Ethical statement

This study was approved by the Medical Research Ethics Review Committee of the General Hospital of Ningxia Medical University, with ethics approval number KYLL-2025-1854. As this is a retrospective study and does not involve any patient-identifying information, the Ethics Committee waived the requirement for written informed consent.

## 3 Results

### 3.1 Clinical and pathological characteristics

The MBC cohort included 27 women aged 39 to 74 years, with a median age of 56 years. Thirteen tumors were in the left breast and 14 in the right breast. Menopausal status was premenopausal in 5 patients, perimenopausal in 1, and postmenopausal in 21. Pathological T stage was T1 in 8 patients (29.6%), T2 in 10 (37.0%), T3 in 5 (18.5%), and T4 in 4 (14.8%). Pathological N stage was N0 in 20 patients (74.1%), N1 in 6 (22.2%), N2 in 0, and N3 in 1 (3.7%). **Table 1** summarizes the clinicopathological characteristics.

**Table 1:** Clinicopathological characteristics of 27 patients with MBC.

Characteristics	Number of patients	Percentage (%)
T stage		
T1	8	29.6
T2	10	37.0
T3	5	18.5
T4	4	14.8
N stage		
N0	20	74.1
N1	6	22.2
N2	0	0.0
N3	1	3.7
ER status		
Positive	1	3.7
Negative	26	96.3
PR status		
Positive	1	3.7
Negative	26	96.3
HER-2 status		
Positive	0	0.0
Negative	27	100.0
Ki-67 (%)		
<30	0	0.0
≥30	27	100.0
Subtypes of MBC		
Squamous cell carcinoma type	14	51.9
Spindle cell carcinoma type	2	7.4
Matrix-producing or osseous-chondroid type	3	11.1
Mixed type	4	14.8
Sarcomatoid type	4	14.8

Immunohistochemistry showed ER positivity in 1 patient (3.7%) and ER negativity in 26 (96.3%). PR status followed the same distribution, and all 27 tumors (100.0%) were HER2-negative. Ki-67 was ≥ 30% in every case, indicating active proliferation. Squamous cell carcinoma was the leading subtype (14, 51.9%), followed by mixed type (4, 14.8%), sarcomatoid type (4, 14.8%), stromal or osteochondral differentiation (3, 11.1%), and spindle cell carcinoma (2, 7.4%) (**Figure 1**). Thus, the cohort mainly consisted of triple-negative tumors with high Ki-67 expression and frequent squamous differentiation.

**Figure 1:**
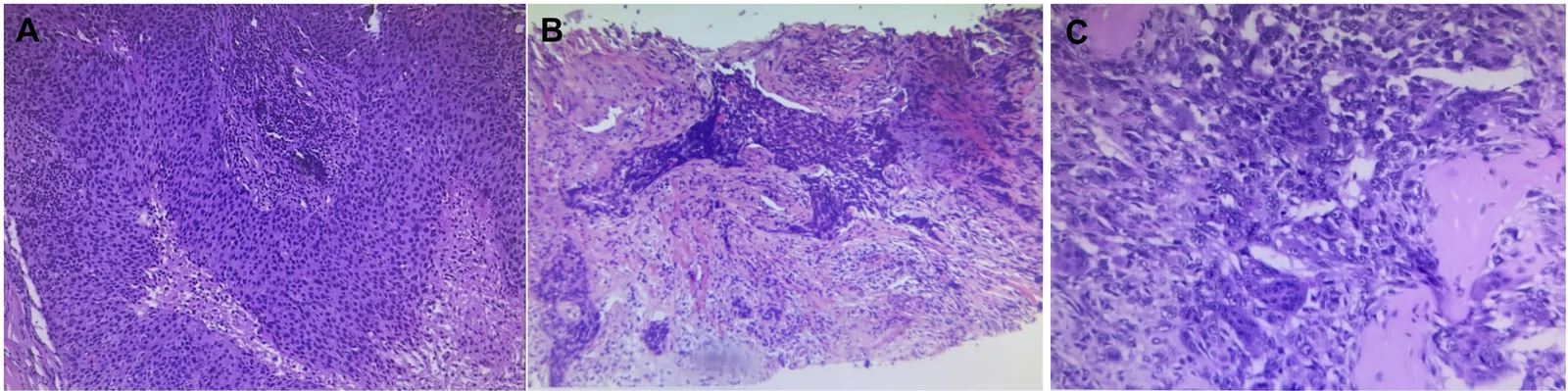
Representative histopathological features of metaplastic breast carcinoma. **(A)** Squamous cell carcinoma type, showing anaplastic tumor cells arranged in nests or sheets with squamous differentiation (HE × 100). **(B)** Chondroblastic or matrix-producing type, showing carcinomatous epithelial components mixed with chondroblastic or mucoid matrix components (HE × 40). **(C)** Spindle cell carcinoma with osteoid differentiation, showing atypical spindle-shaped or pleomorphic tumor cells with osteoid matrix formation (HE × 200).

### 3.2 Comparison of clinical-pathological and immunohistochemical characteristics between the two groups

#### 3.2.1 Comparison of clinical-pathological characteristics

The comparative cohort included 27 patients with MBC and 81 with TNBC (**Table 2**). Age and menopausal status were similar between groups (P = 0.269 and P = 0.128, respectively). MBC showed a higher proportion of grade III tumors (88.9% vs. 28.7%, P < 0.001) and T3–T4 tumors (33.3% vs. 11.1%, P = 0.007). By contrast, lymph node metastasis was less frequent in MBC, with N1 or higher disease in 25.9% of MBC patients and 51.9% of TNBC patients (P = 0.019). M1 disease was numerically more common in MBC, although the difference did not reach statistical significance (14.8% vs. 3.7%, P = 0.064). Overall stage was comparable (P = 0.707). Squamous cell carcinoma was the most common MBC subtype (51.9%), whereas invasive breast carcinoma of no special type dominated the TNBC group (96.3%).

**Table 2:** Comparison of clinicopathological features between MBC and TNBC.

Variables	MBC group (n = 27)	TNBC group (n = 81)	*P* value
Age, years	56.7 ± 9.3	54.2 ± 10.2	0.269
Menopausal status			0.128
Premenopausal	6 (22.2%)	31 (38.3%)	
Postmenopausal	21 (77.8%)	50 (61.7%)	
Histological grade			<0.001
I–II	3 (11.1%)	57 (71.2%)	
III	24 (88.9%)	23 (28.7%)	
T stage			0.007
T1–T2	18 (66.7%)	72 (88.9%)	
T3–T4	9 (33.3%)	9 (11.1%)	
Lymph node status			0.019
N0	20 (74.1%)	39 (48.1%)	
≥N1	7 (25.9%)	42 (51.9%)	
M stage			0.064
M0	23 (85.2%)	78 (96.3%)	
M1	4 (14.8%)	3 (3.7%)	
Tumor stage			0.707
I–II	19 (70.4%)	60 (74.1%)	
III–IV	8 (29.6%)	21 (25.9%)	
Histological subtype of MBC			
Squamous cell carcinoma	14 (51.9%)	—	
Mixed type	4 (14.8%)	—	
Sarcomatoid carcinoma	4 (14.8%)	—	
Chondroid differentiation	2 (7.4%)	—	
Spindle cell carcinoma with osseous differentiation	1 (3.7%)	—	
Spindle cell carcinoma	1 (3.7%)	—	
Osseous-chondroid differentiation	1 (3.7%)	—	
Histological subtype of TNBC			
Invasive carcinoma of no special type	—	78 (96.3%)	
Invasive lobular carcinoma	—	3 (3.7%)	

#### 3.2.2 Comparison of immunohistochemical characteristics

**Table 3** presents the immunohistochemical comparison. One MBC tumor was ER-positive, and one was PR-positive (3.7% each), whereas all TNBC patients were ER- and PR-negative; all tumors in both cohorts were HER2-negative. ER, PR, and HER2 were not statistically compared between groups. CK5/6 and EGFR positivity were common in both cohorts. CK5/6 positivity was 70.4% in MBC and 80.2% in TNBC (*P* = 0.285), while EGFR positivity was 88.9% and 81.5%, respectively (*P* = 0.553). The Ki-67 index was significantly higher in MBC than in TNBC (54.0 ± 16.3% vs. 43.5 ± 22.0%, P = 0.011). High Ki-67 expression, using the 30% cutoff, was also more frequent in MBC (100.0% vs. 80.5%, *P* = 0.010).

**Table 3:** Comparison of immunohistochemical features between MBC and TNBC.

Variables	MBC group (n = 27)	TNBC group (n = 81)	*P* value
ER status			—
ER positive	1 (3.7%)	0 (0.0%)	
ER negative	26 (96.3%)	81 (100.0%)	
PR status			—
PR positive	1 (3.7%)	0 (0.0%)	
PR negative	26 (96.3%)	81 (100.0%)	
HER2 status			—
HER2 positive	0 (0.0%)	0 (0.0%)	
HER2 negative	27 (100.0%)	81 (100.0%)	
CK5/6 expression			0.285
Positive	19 (70.4%)	65 (80.2%)	
Negative	8 (29.6%)	16 (19.8%)	
EGFR expression			0.553
Positive	24 (88.9%)	66 (81.5%)	
Negative	3 (11.1%)	15 (18.5%)	
Ki-67 index, %	54.0 ± 16.3	43.5 ± 22.0	0.011
High Ki-67 expression			0.010
≥30%	27 (100.0%)	65 (80.5%)	
<30%	0 (0.0%)	15 (18.5%)	

### 3.3 Comparison of treatment methods between the two groups

Treatment modalities are compared between the two groups in **Table 4**. Mastectomy-related procedures were the predominant surgical approach in both cohorts. Mastectomy was performed in 92.6% of patients with MBC and 85.2% of patients with TNBC, whereas breast-conserving surgery or local excision was less frequent in MBC (3.7% vs. 14.8%). The distribution of surgical procedures did not differ significantly between groups (P = 0.072). Neoadjuvant chemotherapy was used significantly less often in MBC than in TNBC (11.1% vs. 53.1%, P < 0.001). Among the three patients with MBC who received neoadjuvant systemic therapy, one initially received three cycles of JS001 (toripalimab) plus nab-paclitaxel. JS001 was then discontinued because of hypothyroidism. Nab-paclitaxel was continued alone. Serial response assessments after 2, 4, and 6 cycles showed PR, PD, and SD, respectively. PD was attributed to enlargement of the primary breast lesion. This patient then received six cycles of AC plus thalidomide, with further PD, followed by five cycles of GP (gemcitabine plus carboplatin). Of the remaining two patients, one received six cycles of TCb (paclitaxel plus carboplatin) and achieved SD, whereas the other received six cycles of TP (paclitaxel plus cisplatin) and achieved PR. No pCR was observed among the three patients.

**Table 4:** Comparison of treatment methods between MBC and TNBC.

Treatment method	MBC group (n = 27)	TNBC group (n = 81)	*P* value
Surgical procedure			0.072
Mastectomy	25 (92.6%)	69 (85.2%)	
Breast-conserving	1 (3.7%)	12 (14.8%)	
No surgery	1 (3.7%)	0 (0.0%)	
Neoadjuvant chemotherapy			<0.001
Yes	3 (11.1%)	43 (53.1%)	
No	24 (88.9%)	38 (46.9%)	
Adjuvant chemotherapy			0.147
Yes	20 (74.1%)	70 (86.4%)	
No	7 (25.9%)	11 (13.6%)	
Radiotherapy			0.003
Yes	13 (48.1%)	64 (79.0%)	
No	14 (51.9%)	17 (21.0%)	
Endocrine therapy			—
Yes	2 (7.4%)	0 (0.0%)	
No	25 (92.6%)	81 (100.0%)	

Postoperative adjuvant chemotherapy was administered to 74.1% of patients with MBC and 86.4% of patients with TNBC (P = 0.147). Radiotherapy was also less frequently used in MBC (48.1% vs. 79.0%, P = 0.003). Two patients with MBC received endocrine therapy, whereas no patients with TNBC did; this variable was not statistically compared.

### 3.4 Prognostic analysis and comparison between groups

#### 3.4.1 Recurrence and metastasis

During follow-up, recurrence or metastasis occurred more frequently in patients with MBC than in those with TNBC (25.9% vs. 8.6%, P = 0.041). Local or regional recurrence was uncommon and comparable between the groups (3.7% vs. 6.2%, P = 1.000). In the MBC group, the single local or regional event involved the chest wall or breast, whereas regional nodal recurrence was the main pattern in the TNBC group. Distant metastasis was significantly more frequent in MBC (22.2% vs. 2.5%, P = 0.003). The lung was the most common distant metastatic site in MBC, with liver, brain, and reproductive system or other sites also recorded. In contrast, distant metastatic events were fewer in TNBC and mainly involved the lung and liver (**Table 5**). Because several patients had multiple metastatic sites and the number of events was small, metastatic site distribution was described without additional between-group statistical testing.

**Table 5:** Comparison of recurrence and metastatic patterns between MBC and TNBC.

Recurrence or metastasis pattern	MBC group (n = 27)	TNBC group (n = 81)	*P* value
Overall recurrence or metastasis			0.041
Yes	7 (25.9%)	7 (8.6%)	
No	20 (74.1%)	74 (91.4%)	
Regional recurrence			1.000
Yes	1 (3.7%)	5 (6.2%)	
No	26 (96.3%)	76 (93.8%)	
Chest wall	1 (100.0%)	1 (20.0%)	—
Regional lymph nodes	0 (0.0%)	4 (80.0%)	—
Distant metastasis			0.003
Yes	6 (22.2%)	2 (2.5%)	
No	21 (77.8%)	79 (97.5%)	
Lung	3 (50.0%)	1 (50.0%)	—
Liver	1 (16.7%)	1 (50.0%)	—
Brain	1 (16.7%)	0 (0.0%)	—
Bone	0 (0.0%)	0 (0.0%)	—
Reproductive system or other sites	3 (50.0%)	0 (0.0%)	—

#### 3.4.2 Survival analysis

The median follow-up duration was 72.0 months (95% CI, 48.6–95.0) in the MBC cohort and 71.5 months (95% CI, 50.5–88.4) in the TNBC cohort, with no significant difference between the two groups (*P* = 0.933; **Supplementary Table S2**). Kaplan-Meier analysis indicated a lower DFS curve for MBC than for TNBC, but the difference was not statistically significant (*P* = 0.200). DFS analysis included 23 patients with MBC and 78 with TNBC, and median DFS was not reached in either cohort. OS, in contrast, was poorer in MBC (*P* = 0.038). The OS analysis included all 27 MBC patients and 81 TNBC patients, with median OS not reached in either group. Thus, MBC was linked to worse OS in this cohort, while the DFS comparison remained non-significant (**Figure 2**).

**Figure 2:**
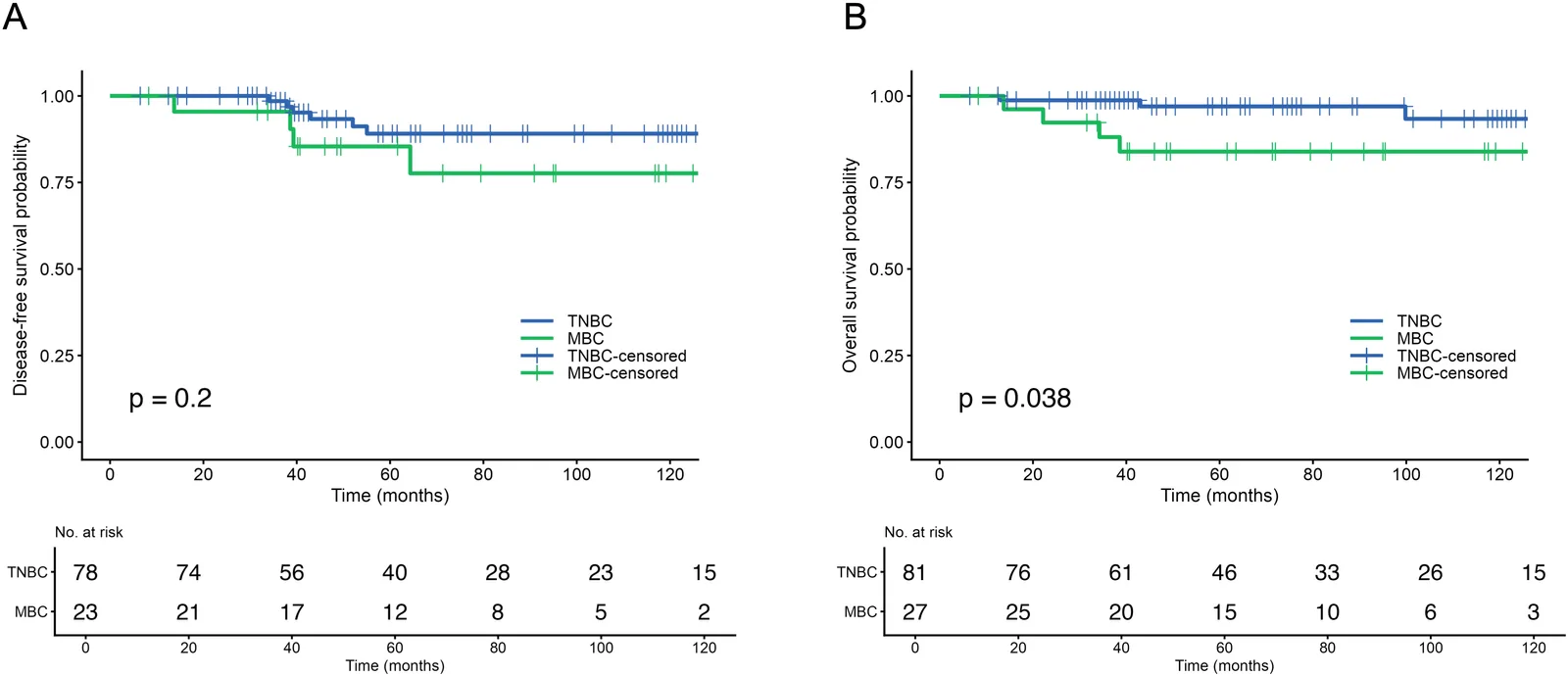
Kaplan-Meier survival curves comparing DFS and OS between patients with MBC and patients with TNBC. **(A)** Disease-free survival. **(B)** Overall survival.

During follow-up, four deaths occurred in the MBC cohort and three in the TNBC cohort. Of these, two deaths in each cohort were attributable to breast cancer progression or distant metastasis. One additional death in the MBC cohort was treatment-related, resulting from chemotherapy-induced myelosuppression complicated by septic shock, while the remaining deaths were due to natural death or unknown causes. Breast cancer-specific deaths occurred in 2 of 27 patients (7.4%) in the MBC cohort and 2 of 81 patients (2.5%) in the TNBC cohort, with no significant difference between the groups (P = 0.260; **Supplementary Table S3**).

### 3.5 Univariate survival analysis of 5-year DFS and 5-year OS in the MBC group

For DFS, N stage, pathological stage, and neoadjuvant chemotherapy were associated with outcomes (**Table 6**). Patients with node-positive disease had a lower 5-year DFS rate than those with N0 disease (66.7% vs. 93.8%, *P* = 0.008). Stage III–IV disease was also linked to lower 5-year DFS than stage I–II disease (33.3% vs. 100.0%, *P* < 0.001). Neoadjuvant chemotherapy was associated with poorer DFS (*P* = 0.005), but this result should be interpreted cautiously because only a small number of DFS-evaluable patients received neoadjuvant treatment. T stage showed a borderline relationship with DFS; patients with T3–T4 disease had lower 5-year DFS than those with T1–T2 disease (60.0% vs. 100.0%, *P* = 0.063).

**Table 6:** Univariate analysis of prognostic factors for DFS and OS in patients with MBC.

Variables	Cases (DFS/OS)	DFS			OS		
		5-year DFS (%)	χ²	*P* value	5-year OS (%)	χ²	*P* value
Age (years)			0.06	0.803		0.05	0.828
<56	9/13	100.0			82.5		
≥56	13/14	83.3			85.1		
Menopausal status			1.53	0.216		1.39	0.238
Premenopausal	6/6	100.0			100.0		
Postmenopausal	16/21	84.6			78.8		
T stage			3.45	0.063		3.20	0.073
T1–2	17/18	100.0			93.3		
T3–4	5/9	60.0			66.7		
N stage			7.08	0.008		1.27	0.261
N0	19/20	93.8			88.8		
N+	3/7	66.7			71.4		
Pathological stage			12.93	<0.001		10.60	0.001
I–II	19/19	100.0			100.0		
III–IV	3/8	33.3			50.0		
Histological subtype			0.16	0.693		0.91	0.341
Squamous type	12/14	100.0			92.3		
Non-squamous type	10/13	80.0			76.9		
Lymphovascular invasion			0.58	0.447		0.83	0.362
Negative	19/23	93.8			80.8		
Positive	3/4	66.7			100.0		
Hormone receptor status			0.11	0.735		0.38	0.540
Negative	21/25	88.9			82.5		
Positive	1/2	100.0			100.0		
Neoadjuvant chemotherapy			7.77	0.005		0.89	0.346
No	21/24	94.4			86.1		
Yes	1/3	0.0			66.7		
Adjuvant chemotherapy			0.10	0.748		1.11	0.292
No	5/7	80.0			71.4		
Yes	17/20	92.9			88.8		
Radiotherapy			0.14	0.710		0.62	0.432
No	11/14	90.9			78.6		
Yes	11/13	87.5			91.7		

For OS, pathological stage was the only statistically significant factor. The 5-year OS rate was lower in stage III–IV disease than in stage I–II disease (50.0% vs. 100.0%, *P* = 0.001). T stage showed a similar but non-significant trend (66.7% vs. 93.3%, *P* = 0.073). Age, menopausal status, histological subtype, lymphovascular invasion, hormone receptor status, adjuvant chemotherapy, and radiotherapy were not significantly associated with DFS or OS.

## 4 Discussion

This study supports the clinical impression that MBC, despite its frequent triple-negative immunophenotype, behaves differently from conventional non-metaplastic TNBC. In the matched comparison, MBC showed higher histological grade, more T3–T4 disease, a higher Ki-67 index, and less nodal involvement. It was also associated with higher rates of recurrence or metastasis, more frequent distant spread, and poorer OS. Taken together, these findings point to an aggressive pattern that is not captured by triple-negative status alone. The large proportion of grade III and T3–T4 tumors suggests that many patients already carried a considerable tumor burden at diagnosis (19). This observation agrees with reports describing MBC as a rapidly enlarging breast mass with high-grade morphology (20). Its metaplastic components, including squamous, spindle-cell, chondroid, and osteoid differentiation, may underlie part of this heterogeneity. In our cohort, squamous cell carcinoma remained the predominant subtype, as reported in several previous series (21).

The survival pattern observed in our cohort was broadly consistent with previous comparisons between MBC and TNBC (16, 20, 22). Previous database analyses and single-center studies have reported inferior survival, including poorer DFS and OS, in patients with MBC compared with TNBC (23, 24). Our cohort also demonstrated poorer OS but did not show a statistically significant DFS difference. Several factors may explain this divergence. The sample size was small, DFS events were limited, and the analysis excluded patients with metastatic disease at presentation and those who did not undergo surgery. These exclusions may have weakened the contrast in early progression risk. Differences in treatment exposure, calendar period, and follow-up intensity could also affect DFS estimates. Therefore, the non-significant DFS comparison should be interpreted alongside the higher distant metastasis rate and poorer OS, rather than as evidence of equivalent disease behavior.

The pattern of spread is one of the more clinically relevant differences observed in this study. MBC was characterized by larger primary tumors, less frequent lymph node involvement, and substantially more frequent distant metastasis (19, 22). This distribution is compatible with earlier descriptions of MBC as a tumor with relatively limited nodal spread but a propensity for hematogenous dissemination. Potential biological contributors include metaplastic differentiation, epithelial-mesenchymal transition, basal-like features, and alterations in pathways such as PI3K/AKT/mTOR and Wnt (25, 26). Importantly, recurrent genomic alterations involving these signaling pathways, particularly the PI3K/AKT/mTOR pathway, may have therapeutic relevance and support further investigation of molecularly guided targeted strategies in selected patients with MBC. CK5/6 and EGFR were frequently positive in both groups, indicating overlap in basal-like marker expression (26). However, the higher Ki-67 index observed in MBC suggests a stronger proliferative component (27). Together, these findings argue that the adverse course of MBC is not simply a consequence of being triple-negative, but may reflect the combined influence of histological diversity, proliferation, and metastatic route.

Treatment-related findings require caution. Neoadjuvant chemotherapy and radiotherapy were used less often in MBC than in TNBC, reflecting both the rarity of the disease and possible differences in presentation or treatment selection (28). In practice, MBC is usually treated according to TNBC or high-risk invasive breast cancer principles because prospective randomized evidence is lacking. Notably, our cohort was treated between 2015 and 2023, a period during which the role and indications of neoadjuvant systemic therapy for TNBC evolved substantially and became progressively more standardized. Therefore, treatment patterns in this real-world cohort also reflect temporal heterogeneity in clinical practice, as well as individualized treatment selection, including patient preference for upfront surgery in a small proportion of cases. Prior studies have reported limited chemotherapy sensitivity, especially in the neoadjuvant setting, and lower pathological complete response rates than in typical TNBC (15, 29). In our cohort, few MBC patients received neoadjuvant treatment, responses were mainly stable disease or partial response, and no definite pathological complete response was observed. The association between neoadjuvant chemotherapy and poorer DFS should therefore be viewed as a marker of baseline risk rather than a harmful treatment effect. Similarly, the absence of a survival association for radiotherapy may reflect indication bias, small numbers, and few events (30). The potential roles of radiotherapy, platinum-based chemotherapy, immunotherapy, and targeted therapy remain important questions for larger studies.

Several limitations should be considered. This was a retrospective, single-center study, and both the MBC cohort and the number of outcome events were small. These features increase the possibility of selection bias and limit the statistical power of prognostic analyses, which should therefore be regarded as exploratory. The TNBC controls were matched by diagnosis year and age, but not by T stage, N stage, or overall stage; residual stage-related confounding is therefore possible. In addition, some immunohistochemical variables were extracted from original pathology reports as positive or negative results, without uniform semi-quantitative scoring. Molecular and immune markers, including programmed death-ligand 1 (PD-L1), tumor-infiltrating lymphocytes (TILs), phosphatidylinositol-4,5-bisphosphate 3-kinase catalytic subunit alpha (PIK3CA), tumor protein p53 (TP53), and Wnt pathway alterations, were unavailable, limiting biological interpretation. Despite these constraints, the contemporaneous TNBC comparison suggests that MBC differs from non-metaplastic TNBC in tumor burden, proliferative activity, distant metastatic risk, and OS. Accurate pathological diagnosis, sufficient tissue sampling, and vigilant assessment for distant disease may therefore be particularly important in the clinical management of MBC.

## 5 Conclusions

In summary, MBC in this cohort differed from conventional non-metaplastic TNBC in clinicopathological profile, metastatic behavior, and survival outcomes. The MBC group showed higher histological grade, greater tumor burden, stronger proliferative activity, less frequent nodal involvement, more frequent distant metastasis, and poorer OS. These findings support the view that MBC should be considered a distinct high-risk entity rather than merely a rare form within the TNBC spectrum. Larger multicenter cohorts incorporating molecular data are needed to improve risk stratification and guide individualized treatment.

## Data Availability

The original contributions presented in the study are included in the article/**Supplementary Material**. Further inquiries can be directed to the corresponding author.
