# Supplementary material for: Metaplastic breast carcinoma versus triple-negative breast cancer: distinct metastatic patterns and inferior overall survival in a matched retrospective cohort study
Source: Front Oncol. 2026 Sep 22;16:1932260. doi: 10.3389/fonc.2026.1932260 (PMC13638616; doi:10.3389/fonc.2026.1932260)
Supplement: Supplementary file 1 [file DataSheet1.zip › Supplementary fiels/Ethic_approval/Ethics Approval KYLL-2025-1854.pdf]

Approval Letter of the Medical Research Ethics Review Committee
General Hospital of Ningxia Medical University

**Project Title**	A Retrospective Matched Cohort Study Comparing the Clinical Characteristics and Prognosis of Metaplastic Breast Carcinoma and Triple-Negative Breast Cancer					**Ethics Approval No.**	KYLL-2025-1854
**Principal Investigator**	Li Jinping	**Professional Title**	Chief Physician	**Telephone**	13995105618	**Department/ Office**	Hospital Administration

**Principal Investigator's Institution**	General Hospital of Ningxia Medical University
**Collaborating Institution**	The First Clinical Medical College of Ningxia Medical University
**Study Period**	November 1, 2025 to June 20, 2027
**Use of Human Tissue Specimens and Samples**	Yes
**Study Sample Size**	100
**Involvement of Stem Cells and Regenerative Medicine**	No
**Source of the Research Project**	Other

Other Ethics-Related Documents

**Informed Consent Form Attached**	1
**Method of Obtaining Informed Consent**	Appropriate

Uploaded Attachments

**Informed Consent Form**	Informed Consent Form of the General Hospital of Ningxia Medical University - Li Jinping.pdf
**Study Protocol**	Study Protocol.pdf
**Technical Roadmap**	Technical Roadmap.pdf
**Presentation Slides**	Medical Research Ethics Approval Application Presentation, General Hospital of Ningxia Medical University - Li Jinping.pdf
**Research Integrity Commitment**	Research Integrity Commitment, General Hospital of Ningxia Medical University - Li Jinping.pdf
**Documentation of the Source of Biological Samples / Information / Data**	
**Conflict-of-Interest Declaration**	
**Recruitment Advertisement and Mode of Publication**	
**Review Type**	01
**Annual / Periodic Continuing Review Frequency**	Every 12 months
**Review Opinion**	The study complies with ethical requirements and may be conducted in accordance with the approved protocol.

Committee Approval Decisions	Committee Member / Decision
Committee Approval Decisions	Niu Zhanfeng **[APPROVED]**
	Wang Youshen **[APPROVED]**
	Ding Xiaoyun **[APPROVED]**
	Zhao Qian **[APPROVED]**
	Wang Yanrong **[APPROVED]**
	Li Xiaorong **[APPROVED]**
	Lu Hongyan **[APPROVED]**
	Zhu Li **[APPROVED]**
	Chen Juan **[APPROVED]**

宁夏医科大学总医院医学科研伦理审查委员会批准书

项目名称	化生性乳腺癌与三阴性乳腺癌临床特征及预后比较的回顾性匹配队列研究			伦理编号	KYLL-2025-1854		
项目负责人	李金平	职称	主任医师	联系电话	13995105618	科室	院部

负责人研究单位：	宁夏医科大学总医院
合作研究单位：	宁夏医科大学第一临床医学院
研究时间：	2025-11-01 至 2027-06-20
是否使用人体组织标本和样本	是
试验数量：	100
是否涉及干细胞与再生医学	否
研究项目来源：	其他
其他伦理相关文件	
是否附有知情同意书：	1
获取知情同意书方法：	适当
附件上传：	
知情同意书	宁夏医科大学总医院知情同意书 李金平.pdf
试验方案	研究方案.pdf
技术路线图	技术路线图.pdf
幻灯PPT	宁夏医科大学总医院医学科研伦理审批申请汇报 李金平.pdf
研究材料诚信承诺书	宁夏医科大学总医院科研诚信承诺书-李金平.pdf
生物样本信息数据的来源证明	
利益冲突申明	
招募广告及其发布形式	
审查形式	01
年度/定期跟踪审查频率	十二个月
评审意见：	符合伦理学要求，可以按照此方案进行试验。

医学科研伦理委员会审核通过

宁夏医科大学总医院医学科研伦理审查委员会

审批时间：2025年10月22日

委员会审批意见	牛占锋 【通过】<br>王幽深 【通过】<br>丁晓云 【通过】<br>赵倩 【通过】<br>王艳荣 【通过】<br>李晓荣 【通过】<br>芦鸿雁 【通过】<br>朱力 【通过】<br>陈娟 【通过】

医学科研伦理委员会审核通过
